# Designing the Interface Layer of Solid Electrolytes for All‐Solid‐State Lithium Batteries

**DOI:** 10.1002/advs.202401453

**Published:** 2024-06-03

**Authors:** Qian Xia, Shuoguo Yuan, Qiang Zhang, Can Huang, Jun Liu, Hongyun Jin

**Affiliations:** ^1^ Faculty of Materials Science and Chemistry China University of Geosciences Wuhan 430074 China; ^2^ Guangdong Provincial Key Laboratory of Advanced Energy Storage Materials School of Materials Science and Engineering South China University of Technology Guangzhou 510641 China

**Keywords:** interface, LATP, MoS_2_, solid‐state electrolytes, solid‐state lithium batteries

## Abstract

Li_1.3_Al_0.3_Ti_1.7_(PO_4_)_3_ (LATP) is one of the most attractive solid‐state electrolytes (SSEs) for application in all‐solid‐state lithium batteries (ASSLBs) due to its advantages of high ionic conductivity, air stability and low cost. However, the poor interfacial contact and slow Li‐ion migration have greatly limited its practical application. Herein, a composite ion‐conducting layer is designed at the Li/LATP interface, which a MoS_2_ film is constructed on LATP via chemical vapor deposition, followed by the introduction of a solid polymer (SP) liquid precursor to form a MoS_2_@SP protective layer. This protective layer not only achieves a lower Li‐ion migration energy barrier, but also adsorbs more Li‐ion, which is able to promote interfacial ion transport and improve interfacial contacts. Thanks to the improved migration and adsorption of Li‐ion, the Li symmetric cell containing LATP‐MoS_2_@SP exhibits a stable cycle of more than 1200 h at 0.1 mA cm^−2^. More remarkably, the capacity retention of the full cell assembled with LiFePO_4_ cathode is as high as 86.2% after 400 cycles at 1 C. This work provides a design strategy for significantly improving unstable interfaces of SSEs and realizing high‐performance ASSLBs.

## Graphical abstract

A composite ion‐conducting protective layer of MoS_2_/solid polymer is designed onto Li_1.3_Al_0.3_Ti_1.7_(PO_4_)_3_ (LATP), improving its poor Li/LATP interfacial contact and slow Li‐ion migration issues, which obtains excellent electrochemical performance. This study emphasizes the importance of promoting ion migration and adsorption in improving the interface, confirmed by density functional theory calculations and a series of experimental characterizations.

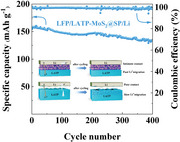

## 1 Introduction

In many energy storage systems, lithium‐based batteries are gradually replacing lead‐acid batteries and nickel‐metal hydride batteries by virtue of their advantages of high energy density, high operating voltage, long cycle life, and stable discharge performance, which have been widely used in the fields of electric energy storage, automobile transportation, and portable devices.^[^ ^1^ ^]^ For safety performance and energy density considerations, lithium‐based batteries are gradually developing from liquid to solid state, of which all‐solid‐state lithium‐metal batteries (ASSLBs) not only solve the safety problems of liquid Li‐ion batteries but also realize a higher specific energy battery system, which is considered to be the most promising candidate for the next generation of green energy storage systems.^[^ ^2^ ^]^ Solid‐state electrolytes (SSEs), as a key component of ASSLBs, typically determine the electrochemical performance of batteries. Among these SSEs, the NASICON‐type solid electrolyte Li_1.3_Al_0.3_Ti_1.7_(PO_4_)_3_ (LATP) with relatively high room temperature Li ionic conductivity (≈10^−3^ S cm^−1^), good chemical stability, and low raw material price is considered to be one of the most attractive liquid electrolyte alternatives.^[^ ^3^ ^]^ Despite these advantages, the instability of the Li‐metal anode, which is widely present in NASICON‐type electrolytes, has limited their development.^[^ ^4^ ^]^

Due to the strong reducibility of Li‐metal, LATP will undergo a side reaction when in contact with Li‐metal, in which Ti^4+^ in LATP is reduced to Ti^3+^, thus generating a mixed conductive layer on the surface of LATP.^[^ ^5^ ^]^ The generation of the mixed conductive layer will further promote the side reaction and eventually lead to battery failure.^[^ ^6^ ^]^ Meanwhile, the contact between LATP and electrode is a solid‐solid contact, and this insufficient contact will lead to a great interfacial impedance, thus affecting the application of LATP in ASSLBs. To cope with these challenges, various solutions have been proposed in recent years, including interfacial protective layers,^[^ ^7^ ^]^ SSE structure modification,^[^ ^8^ ^]^ and the addition of liquid electrolytes.^[^ ^9^ ^]^ Among them, constructing an interfacial protective layer of Li/LATP not only avoids the direct contact between Li‐metal and LATP, solving the chemical/electrochemical reaction at the Li/LATP interface but also helps to promote the migration of Li‐ion at the interface, which has a great potential for application.^[^ ^10^ ^]^ So far, a variety of materials such as Al_2_O_3_,^[^ ^11^ ^]^ BN,^[^ ^12^ ^]^ ZnO,^[^ ^13^ ^]^ Al‐LiF,^[^ ^14^ ^]^ PEO‐PAN,^[^ ^15^ ^]^ BN‐based release agent,^[^ ^16^ ^]^ Al/solid polymer electrolyte^[^ ^17^ ^]^ and so on, have been used for Li/LATP interfacial modification. However, the above strategies mainly focus on protecting the SSE from Li‐metal reduction, which can significantly reduce the decomposition of the SSE, but the improvement of Li‐ion migration and adsorption at the interface is relatively limited. Under long‐term cycling, due to the unstable interfacial ion migration, Li‐ion cannot be transported fast enough thus creating voids at the interface leading to deterioration of the Li/LATP interface.^[^ ^18^ ^]^ The interfacial problem between LATP and Li‐metal still exists, and achieving stable interfacial contacts for ASSLBs remains challenging. Therefore, focusing only on protecting the SSE from decomposition is not sufficient, it is necessary to introduce phases with high ionic conductivity and stabilized interfacial contacts at the Li/LATP interface, which can significantly improve the interfacial ion transfer kinetics. However, the study on this aspect in ASSLBs is highly limited.

Herein, we design a MoS_2_ film on the LATP surface grown by chemical vapor deposition (CVD), followed by the introduction of a solid polymer (SP) liquid precursor consisting of poly(ethylene oxide) (PEO) and lithium bis(trifluoromethylsulfonyl)imide (LiTFSI), to form the MoS_2_@SP composite ion‐conductive protective layer. The 1T metal phase of MoS_2_ has high conductivity (10–100 S cm^−1^), which possesses great potential in promoting Li‐ion migration. Moreover, the presence of SP at the interface can adsorb more Li‐ion, which in turn improves the interfacial contact. The MoS_2_@SP composite ion‐conductive protective layer cannot only protect SSE from Li‐metal reduction but also realize a lower migration barrier and higher adsorption energy. The results present that the optimized LATP‐MoS_2_@SP lithium symmetric cell can be cycled for more than 1200 h at 0.1 mA cm^−2^. Furthermore, the LiFePO_4_(LFP)/Li full cell can provide an initial discharge specific capacity of 154.9 mAh g^−1^ at 1 C, and the capacity retention rate is as high as 86.2% after cycling 400 cycles at 60 °C. This work provides a universal design strategy for interfacial modification of ASSLBs to realize high‐performance long‐term cycling.

## 2 Results and Discussion

**Figure 1:**
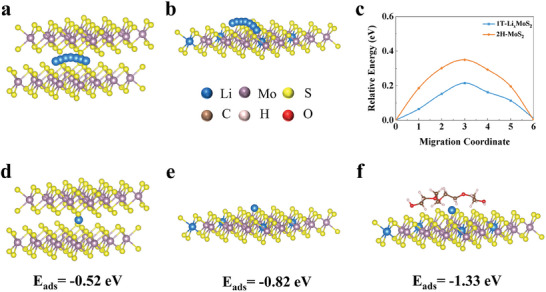
Migration pathways of Li in a) 2H‐MoS_2_ and b) 1T‐Li*_x_* MoS_2_. c) Migration energy barriers for Li in 2H‐MoS_2_ and 1T‐Li*_x_* MoS_2_. Adsorption energy and adsorption structure of d) 2H‐MoS_2_, e) 1T‐Li*_x_* MoS_2_, f) 1T‐Li*_x_* MoS_2_/PEO adsorbed Li.

Typically, it is well known that MoS_2_ exists in morphologies such as 2H‐phase and 1T‐phase depending on the different arrangement of S atoms, 2H‐MoS_2_ has semiconducting properties, whereas 1T‐MoS_2_ is a metallic conductor with a conductivity that is 10^7^ times higher than that of 2H‐MoS_2_, which is due to the incomplete filling of the 4*d* orbital of Mo in 1T‐MoS_2_.^[^ ^19^ ^]^ The high conductivity of metallic 1T‐MoS_2_ makes it more suitable for application at the Li/LATP interface to facilitate Li‐ion transport.^[^ ^20^ ^]^ To verify the claims, 1T‐Li*_x_* MoS_2_ and 2H‐MoS_2_ surfaces (Figure S1a,b, Supporting Information) were chosen as a comparison, and the migration paths of Li‐ion on the 1T‐Li*_x_* MoS_2_ and 2H‐MoS_2_ surfaces were explored by density functional theory (DFT) calculations. The migration energy barriers of Li‐ion on the 1T‐Li*_x_* MoS_2_ and 2H‐MoS_2_ surfaces are 0.22 and 0.35 eV, respectively (**Figure** **1**a–c). The energy barriers of Li‐ion needed to overcome to migrate on the surface of the metallic phase 1T‐Li*_x_* MoS_2_ are much smaller than those of the semiconducting phase 2H‐MoS_2_ (0.13 eV), which can promote diffusive migration of Li‐ion. In addition, the adsorption structures and adsorption energies of Li adsorbed in 2H‐MoS_2_, 1T‐Li*_x_* MoS_2_, and 1T‐Li*_x_* MoS_2_/PEO were further calculated by DFT, the structure of 1T‐Li*_x_* MoS_2_/PEO is shown in Figure S1c (Supporting Information). The adsorption energies of Li adsorbed in 2H‐MoS_2_, 1T‐Li*_x_* MoS_2_, and 1T‐Li*_x_* MoS_2_/PEO are −0.52, −0.82, −1.33 eV, with larger negative values signifying stronger bonding between Li and it (Figure 1d–f). These calculated results demonstrate that 1T‐Li*_x_* MoS_2_ can effectively promote the Li‐ion transport, especially when compounded with PEO, more Li‐ion can be adsorbed. As a result, the MoS_2_ composited with SP liquid precursor consisting of PEO is designed to improve the interface of SSEs, which will achieve high‐performance ASSLBs.

**Figure 2:**
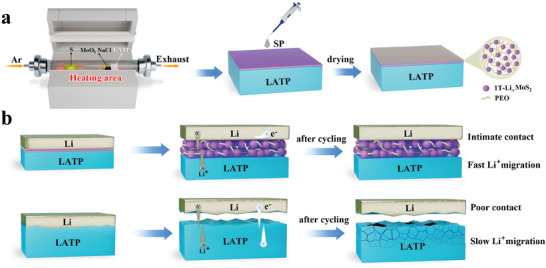
a) Schematic illustration of the preparation process of LATP‐MoS_2_@SP. b) Schematic illustration of interfacial contact and ion migration with and without MoS_2_@SP protective layer, which effectively inhibit the side reactions.

**Figure 3:**
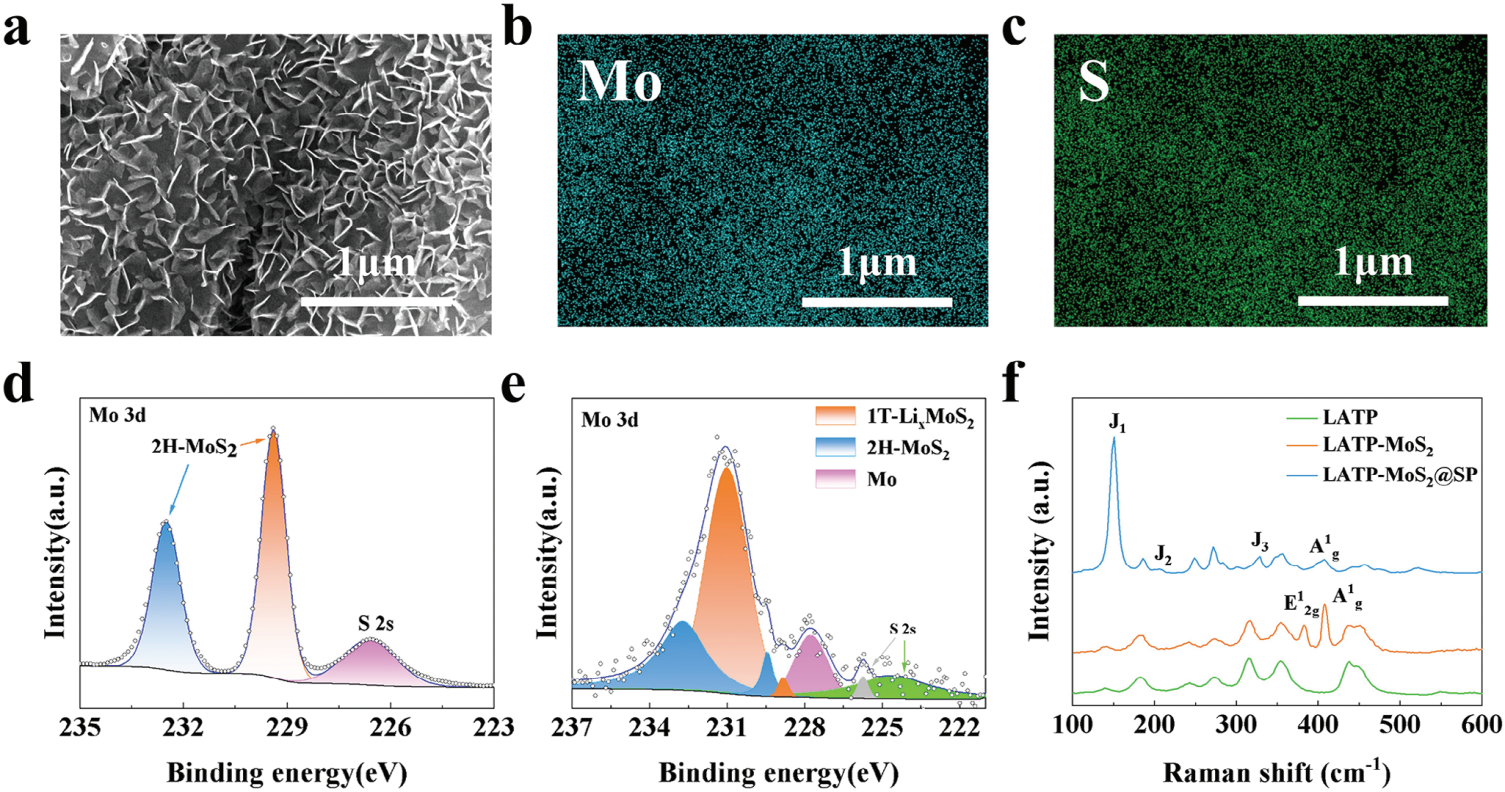
a) Top‐view SEM image and b,c) the corresponding EDS elemental mappings of LATP‐MoS_2_. The Mo 3d XPS spectra of d) LATP‐MoS_2_ and e) LATP‐MoS_2_@SP. f) Raman spectra of LATP, LATP‐MoS_2_, LATP‐MoS_2_@SP.

Based on the above DFT calculations, we choose LATP as an example in this study. The LATP pellets were prepared by solid‐phase reaction method. The X‐ray diffraction (XRD) and scanning electron microscopy (SEM) results display that we have produced pure‐phase LATP with very high densification (Figure S2a,b, Supporting Information). The prepared LATP has a room temperature ionic conductivity of 2.9 × 10^−4^ S cm^−1^ and an activation energy of 0.33 eV (Figure S2c,d, Supporting Information), which gives good electrochemical properties. As shown in **Figure** **2**a, the MoS_2_ thin film was grown using CVD, and a lavender layer was obtained on the LATP surface (Figure S3, Supporting Information). Subsequently, a solid polymer (SP) liquid precursor consisting of PEO and LiTFSI was uniformly coated on the surface of the LATP‐MoS_2_ and then dried in an oven at 60 °C to obtain the modified LATP (named LATP‐MoS_2_@SP). The intermediate layer of MoS_2_@SP not only promotes the migration of Li‐ion but also reduces the interfacial impedance and improves the interfacial contact of Li/LATP (Figure 2b). Figure S4a (Supporting Information) reflects the XRD patterns of LATP‐MoS_2_ and LATP‐MoS_2_@SP, there is no obvious change and shift compared with LATP, this is due to the thickness of the grown MoS_2_ film and SP modification layer, further characterizations are needed to be carried out to prove the existence of the modification layer on the surface of LATP. The SEM images of LATP‐MoS_2_ pellets parade that a large number of fine fibrous flakes of triangular morphology were grown on the LATP surface (**Figure** **3**a and Figure S4b, Supporting Information). The energy dispersive spectrometer (EDS) mapping corresponding to Figure 3a is demonstrated in Figure 3b,c, it reveals that a large amount of Mo and S elements are present on the flake‐like triangular morphology with a homogeneous distribution. Besides, Figure S4c (Supporting Information) displays a cross‐section of the modified LATP‐MoS_2_@SP with a 19 µm interfacial protective layer. X‐ray photoelectron spectroscopy (XPS) measurements were performed on LATP‐MoS_2_ and LATP‐MoS_2_@SP (Figure 3d). In the Mo 3d spectra of LATP‐MoS_2_, the Mo 3d_5/2_ and Mo 3d_3/2_ peaks located at 232.5, 229.4 eV are observed, which represent 2H‐MoS_2_, 226.6 eV peak belongs to the S 2s in 2H‐MoS_2_. The peaks at 163.4 and 162.3 eV in the S 2p spectra of Figure S5a (Supporting Information) further confirm the presence of 2H‐MoS_2_. However, when SP was added, the peaks in the Mo 3d spectra of LATP‐MoS_2_@SP between 226 and 236 eV could be back‐convoluted into five peaks by fitting, with the orange peaks at 231.02 and 228.83 eV, representing 1T‐Li*_x_* MoS_2_, and the blue peaks at 232.72 and 229.46 eV, representing 2H‐MoS_2_. The purple peak at 227.76 eV represents Mo metal, while the peaks at 224.61 and 225.74 eV belong to the S 2s features of Li_2_S and 1T‐Li*_x_* MoS_2_, respectively, indicating that with the insertion of Li‐ion after the addition of SP, the 2H‐MoS_2_ on the surface of the LATP underwent a phase transition and the main phase changes to 1T‐Li*_x_* MoS_2_ with the simultaneous Mo and Li_2_S production (Figure 3e).^[^ ^21^ ^]^ In the S 2p spectra of LATP‐MoS_2_@SP, peaks at 168.9 and 166.4 eV are common in solid electrolyte interfacial studies of LiTFSI salts,^[^ ^22^ ^]^ and the peak at 163.4 eV is attributed to MoS_2_ (Figure S5b, Supporting Information). In the C 1s spectrum, the peaks at 286.6 eV (C─O) and 288.6 eV (C═O) correspond to PEO, and the peak at 293 eV corresponds to LiTFSI (Figure S5c, Supporting Information).^[^ ^22^ ^]^ Raman results exhibit that there are two strong vibrational signals in the Raman spectrum of LATP‐MoS_2_, located at 383 and 408 cm^−1^, which are the characteristic peaks of the rigid vibration E^1^_2g_ and the in‐plane vibration A_1g_ of 2H‐MoS_2_, respectively.^[^ ^23^ ^]^ The Raman peaks of LATP‐MoS_2_@SP, on the other hand, reinforce that the structural crystalline shape of 2H‐MoS_2_ is changed after the addition of SP, i.e., the E^1^_2g_ peak located at 383 cm^−1^ disappears, and new peaks appear at 150, 206, and 328 cm^−1^, corresponding to the J_1_, J_2_, and J_3_ peaks, respectively (Figure 3f). These new peaks are associated with the symmetry reduction of the 1T‐Li*_x_* MoS_2_ phase.^[^ ^24^ ^]^ The whole process can be represented as two processes by the following reactions^[^ ^25^ ^]^:

(1) $$2H-{\mathrm{MoS}}_{2}+{x\mathrm{Li}}^{+}+{xe}^{-}=1T-{\mathrm{Li}}_{x}{\mathrm{MoS}}_{2}\left(x=3∼4\right)$$

(2) $$1T-{\mathrm{Li}}_{x}{\mathrm{MoS}}_{2}+4{\mathrm{Li}}^{+}+4e^{-}=2{\mathrm{Li}}_{2}S+{\mathrm{Mo}/\mathrm{Li}}_{y}$$

To reflect the influence of the protective layer on the interfacial resistance, the electrochemical impedance of the fresh Li/LATP/Li, Li/LATP‐MoS_2_/Li, Li/LATP‐SP/Li, Li/LATP‐MoS_2_@SP/Li symmetric cells at 60 °C were measured. Figure S6a (Supporting Information) displays that the impedance of the Li/LATP/Li cell is as high as 3083.59 Ω. The Li/LATP‐MoS_2_/Li cell and Li/LATP‐SP/Li cell are also as high as 1403.71 and 1538.17 Ω, respectively. In comparison, the impedance of the Li/LATP‐MoS_2_@SP/Li cell is only 725.26 Ω. The results designate that the MoS_2_@SP interlayer can diffuse into the LATP electrolyte to effectively enhance the initial interfacial contact and reduce the interfacial impedance. In addition, cyclic voltammetry (CV) tests were carried out, and the CV results of the full cell assembled by LATP and LATP‐MoS_2_@SP are shown in Figure S6b,c (Supporting Information). The C–V curves of the three cycles are stable, and the redox peaks of the cell appeared near 3.7 and 3.1 V, which are consistent with those of the LFP.^[^ ^26^ ^]^ Besides, no other redox peaks are detected, which proves that the MoS_2_@SP has excellent interfacial stability as a protective layer for LATP.

**Figure 4:**
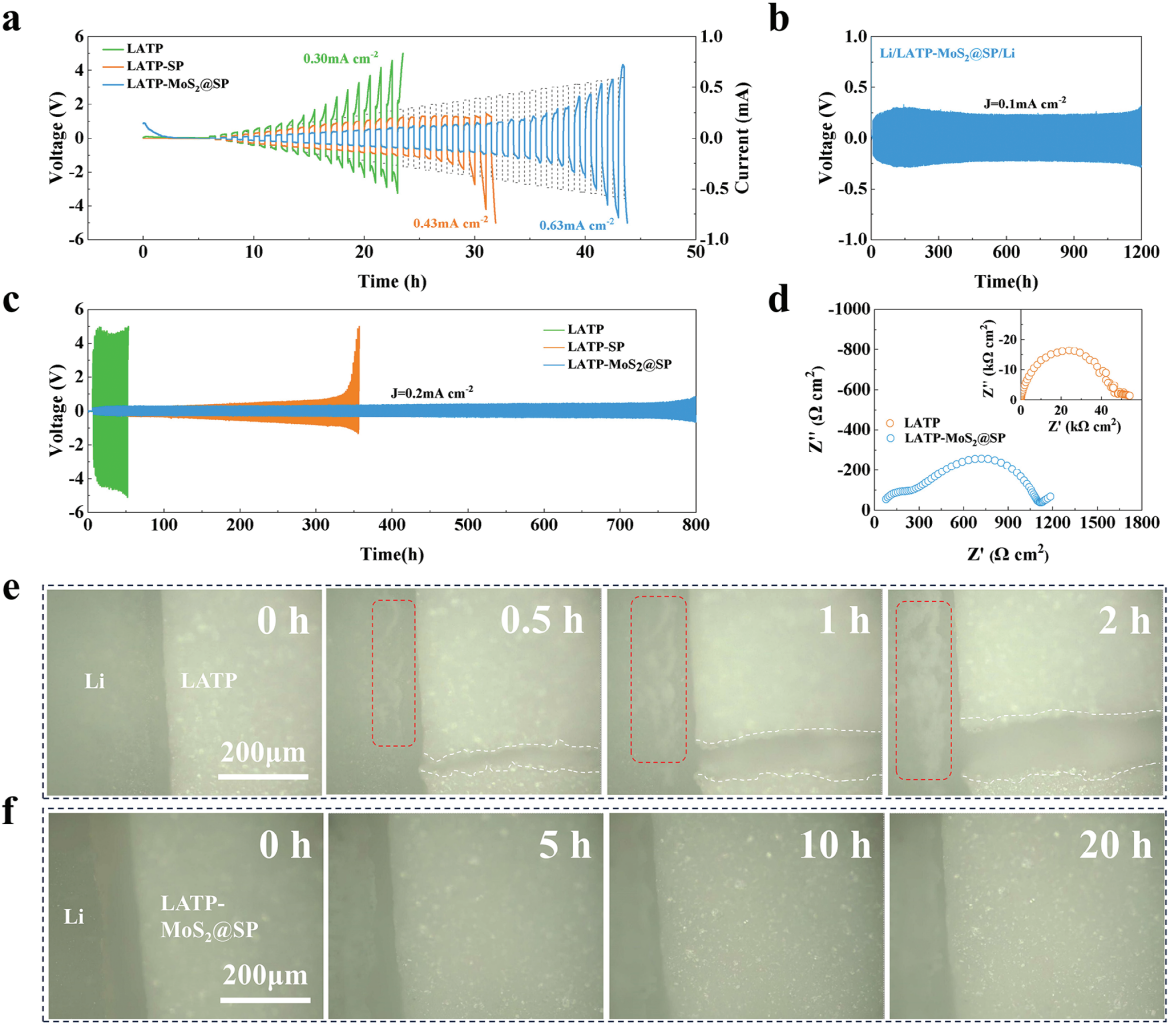
a) CCD measurements of Li/LATP/Li, Li/LATP‐SP/Li, Li/LATP‐MoS_2_@SP/Li cells. b) Constant current stripping and plating of Li/LATP‐MoS_2_@SP/Li cells at 0.1 mA cm^−2^. c) Constant current stripping and plating of Li/LATP/Li, Li/LATP‐SP/Li, Li/LATP‐MoS_2_@SP/Li cells at 0.2 mA cm^−2^. d) Impedance spectra of Li/LATP/Li and Li/LATP‐MoS_2_@SP/Li cells after cycling under 0.05 mA cm^−2^, where Li/LATP/Li cells are cycled for 100 h and Li/LATP‐MoS_2_@SP/Li cells are cycled for 2000 h. Optical analysis of in situ Li plating. Morphological evolution of e) Li/LATP interface and f) Li/LATP‐MoS_2_@SP interface at 0.3 mA cm^−2^ under various times.

Interfacial tolerance is a prerequisite for batteries to be able to operate at high‐current density densities. To investigate the interfacial tolerance between MoS_2_@SP‐modified LATP and Li‐metal, the corresponding Li/LATP‐MoS_2_@SP/Li symmetric cells were assembled. In addition, we also assembled Li/LATP/Li and Li/LATP‐SP/Li symmetric cells as comparison samples, respectively. The critical current density (CCD) test at 60 °C describes that the CCD of the Li/LATP‐MoS_2_@SP/Li symmetric cell is 0.63 mA cm^−2^, whereas that of the Li/LATP/Li and Li/LATP‐SP/Li symmetric cells are 0.30 and 0.43 mA cm^−2^, respectively, which designate that the metallic 1T‐Li*_x_* MoS_2_ modification contributes to the interfacial stability (**Figure** **4**a). Stripping/plating cycle tests were performed subsequently to evaluate the durability of the MoS_2_@SP interlayer. The Li/LATP‐MoS_2_@SP/Li symmetric cell can be stably cycled for 500 h at a current density of 0.05 mA cm^−2^ and then 500 h at a current density of 0.1 mA cm^−2^ with the lowest polarization voltage (Figure S7a, Supporting Information). In contrast, a symmetric cell assembled with bare LATP under the same conditions has failed within 150 h (Figure S7b, Supporting Information). The polarization voltage of the Li/LATP‐SP/Li cells starts to increase significantly after 800 h and reaches 0.25 V after 1000 h. Furthermore, the long‐term stripping/plating curve at a current density of 0.05 mA cm^−2^ presents that the Li/LATP‐MoS_2_@SP/Li cells can be stably cycled for 2000 h or more, with a stable polarization voltage < 0.1 V (Figure S7c, Supporting Information). When the current density is increased to 0.1 mA cm^−2^, the Li/LATP‐MoS_2_@SP/Li cells can cycle stably for 1200 h after a short activation process. (Figure 4b). However, the Li/LATP‐SP/Li cells have failed within 1000 h (Figure S7d, Supporting Information). To further reveal the modification effect of the modification layer on the Li/LATP interface, we performed a comparative test at a high current density of 0.2 mA cm^−2^ (Figure 4c). The electroplating/peeling cycle of the Li/LATP‐MoS_2_@SP/Li cells has a high degree of stability, and no serious damage occurs within 800 h of cycling. In contrast, Li/LATP/Li cells without an intermediate layer not only have a high polarization voltage of 4–5 V, but also have a serious side reaction due to the direct contact between LATP and Li‐metal, and the cells open after 50 h (Figure S7e, Supporting Information). Under the same conditions, the polarization voltage of Li/LATP‐SP/Li cells with only a single intermediate layer also starts to increase dramatically after 300 h of cycling, and the cells fail after 356 h. These results indicate that after the interfacial modification by MoS_2_@SP, the Li/LATP interface parades less Li‐ion migration resistance, resulting from the formation of a layer of stable Li‐ion transport channels at the interface by metallic 1T‐Li*_x_* MoS_2_, which promotes Li‐ion transport and inhibits cleavage of LATP at the same time, especially at high current densities.

The changes in interfacial resistance before and after MoS_2_@SP modification were compared by EIS, and the Nyquist plots of Li/LATP/Li and Li/LATP‐MoS_2_@SP/Li cells after cycling at 0.05 mA cm^−2^ are depicted in Figure 4d. Due to the poor interfacial contact, the impedance of Li/LATP/Li cells reaches 44.4 kΩ after cycling for 100 h. However, the impedance of the Li/LATP‐MoS_2_@SP/Li cells is only 1117.06 Ω even after cycling for 2000 h. This designates that the MoS_2_@SP interlayer greatly reduces the interfacial resistance and improves ion transportation. Figure S8 (Supporting Information) displays the SEM images of the LATP surface of Li/LATP‐MoS_2_@SP/Li and Li/LATP/Li symmetric cells after 50 h of cycling at 0.2 mA cm^−2^, which in turn further validates the role of the MoS_2_@SP interfacial interlayer. The digital photographs reflect that cracks and a large number of black masses are distributed on the surface of LATP disassembled from Li/LATP/Li cells, implying that a serious side reaction has occurred in the contact between LATP and Li leading to LATP cleavage. In contrast, the electrolyte disassembled from the Li/LATP‐MoS_2_@SP/Li cells pinpoint no cracks on the surface and relatively few black clumps, and the SEM images show that the LATP grains are tightly stacked, and a small amount of MoS_2_ is deposited on the surface, which confirm that the metallic 1T‐Li*_x_* MoS_2_ modification layer can effectively protect the LATP from decomposition.

To study the effect of MoS_2_@SP composite ion‐conducting layer on the Li/LATP interface modification more intuitively. We have observed the Li plating behavior of the Li/LATP interface and Li/LATP‐MoS_2_@SP interface during cycling using in situ optical microscopy to monitor the morphological evolution of the interface. A customized Li symmetric cell was assembled for cycling at 0.3 mA cm^−2^. Before cycling, both can be seen to have relatively flat interfaces, and the void between the Li/LATP‐MoS_2_@SP interface is due to the presence of MoS_2_@SP. After starting the cycling, the bare LATP appeared cracks in a short time and gradually increased with cycling. The Li‐metal surface on the other side also appeared uneven dendrites during cycling, indicating that the interface has deteriorated (Figure 4e). In contrast, no cracks and dendrites were observed in Li/LATP‐MoS_2_@SP. Meanwhile, the MoS_2_@SP composite ion‐conducting layer helps to induce uniform deposition of Li, which can be recognized by the increased thickness of Li (Figure 4f).

**Figure 5:**
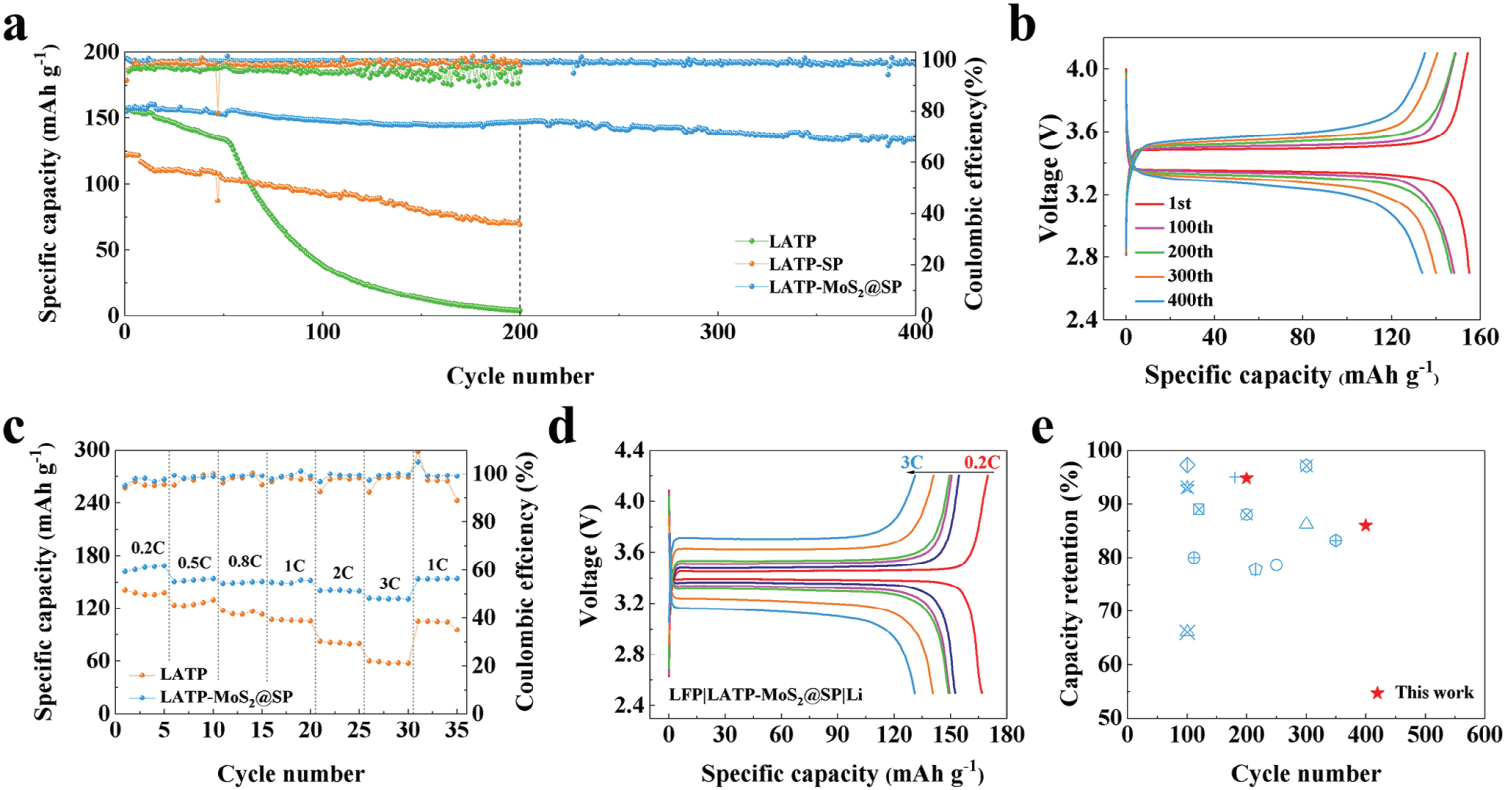
a) Long‐term cycle performance of the LFP/LATP/Li, LFP/LATP‐SP/Li, LFP/LATP‐MoS_2_@SP/Li cells at 60 °C and 1 C. b) The charging/discharging profiles with different cycles in LFP/LATP‐MoS_2_@SP/Li cells. c) Rate performances of the LFP/LATP/Li and LFP/LATP‐MoS_2_@SP/Li cells. d) Corresponding charge‐discharge profiles of the LFP/LATP‐MoS_2_@SP/Li cells. e) Comparison of long‐term cycling performance with previously reported results of LATP solid electrolyte.

The LFP/LATP‐MoS_2_@SP/Li full cells were assembled with LFP as the cathode and Li‐metal as the anode, which were tested for long‐term cycle performance to verify the practicality of MoS_2_@SP protective layer modification of LATP. The electrolyte was sandwiched between the Li‐metal anode and the LFP cathode sheet, while 5 µL of liquid electrolyte (1 M LiPF_6_ in EC: DMC = 1:1) was added dropwise between the electrolyte and the cathode sheet to obtain a more stable interfacial contact. To illustrate the necessity of MoS_2_@SP co‐modification, LFP/LATP/Li and LFP/LATP‐SP/Li full cells were also assembled, and the assembled full cells were subjected to long‐cycle charge/discharge experiments at 60 °C and 1 C (1 C = 170 mA g^−1^). The LFP/LATP‐MoS_2_@SP/Li full cells can provide an initial discharge‐specific capacity of 154.9 mAh g^−1^, capacity retention of 94.8% after 200 cycles, and Coulombic efficiency close to 100% (**Figure** **5**a). Since there will be additional “fresh” Li^+^ stripped from the Li‐metal “reservoir” during the discharge process, resulting in the amount of Li^+^ returned to the cathode being greater than the amount of Li^+^ detached from the cathode during the charging process, hence the Coulombic efficiency of the first cycle is higher than 100%.^[^ ^27^ ^]^ In contrast, the capacity retention of LFP/LATP/Li and LFP/LATP‐SP/Li cells are only 0.3% and 57.1%, respectively, after cycling for 200 cycles under the same conditions. Due to the LFP/LATP‐SP/Li cells having only a bare polymer protective layer, the ionic conductivity is low and the electrochemical stability is poor, which makes its specific capacity lower than other cells. The Coulombic efficiency of the LFP/LATP/Li cells has relatively large randomness over 200 cycles, which can result in interfacial instability that produces a large number of cracks and Li dendrites, leading to additional charging capacity (Figure S9a, Supporting Information).^[^ ^28^ ^]^ Moreover, the capacity retention of the LFP/LATP‐MoS_2_@SP/Li full cells is still as high as 86.2% after 400 cycles, indicating that the full cells with metallic 1T‐Li*_x_* MoS_2_ modified LATP as the electrolyte exhibit excellent cycling stability (Figure 5b). The rate performances of the LFP/LATP‐MoS_2_@SP/Li and LFP/LATP/Li full cells are addressed in Figure 5c,d. The Li/LATP‐MoS_2_@SP/Li full cells at 0.2 C, 0.5 C, 0.8 C, 1 C, 2 C, and 3 C have discharge capacities of 166.7, 152.4, 149.5, 148.9, 140.5, and 130.9 mAh g^−1^, respectively, which are higher than those of LFP/LATP/Li under the same conditions. The discharge capacity of the LFP/LATP‐MoS_2_@SP/Li full cells even recovers to 153.7 mAh g^−1^ when the rate is restored to 1 C, which can be owed to the slower activation process of the cells. The lower capacity of the LFP/LATP/Li full cells at a low rate confirms that LATP is less chemically stable to Li‐metal, resulting in higher interfacial resistance. At a higher rate, the capacity decay is even more pronounced due to the poorer electron/ion dynamics at the Li/LATP interface (Figure S9b, Supporting Information). Measuring the resistance of the full cells after 250 cycles, the equivalent circuit model is shown in Figure S10a (Supporting Information), the resistance of the LFP/LATP/Li cells increases from 108.6 to 4054.1 Ω due to the severe side reactions, whereas the resistance of the LFP/LATP‐MoS_2_@SP/Li cells only increases from 177.5 to 379.8 Ω due to the good interfacial contact (Figure S10b,c, Supporting Information). In the full cells, we have added a liquid electrolyte between the electrolyte and LFP cathode, which could penetrate into the interface between the electrolyte and Li‐metal anode. Therefore, with the help of the liquid electrolyte, the full cells have a much lower impedance than the symmetrical cells. The fresh LFP/LATP‐MoS_2_@SP/Li cells have a slightly higher impedance than the fresh LFP/LATP/Li cells, which may be related to the decomposition of LATP, owing to the lack of a protective layer of LATP in LFP/LATP/Li, the liquid electrolyte penetrates more easily into the electrolyte‐anode interface, resulting in a lower initial impedance. However, during long‐term contact, undesirable interfacial reactions can deteriorate the charge transfer process and lead to cell failure. Further, XRD characterization pinpoints that the crystal phase structure of LATP‐MoS_2_@SP remains intact after 250 cycles, while bare LATP becomes severely damaged (Figure S11, Supporting Information). Figure 5e compares the LFP/Li cells in the present work with that of various reported Li/LATP interfacial modifications in terms of long‐term cycle performance, corresponding to the references in Table S1 (Supporting Information).^[^ ^29^ ^]^ The electrochemical performance of the LFP/Li full cells obtained by modifying the Li/LATP interface with MoS_2_@SP outperforms previous results, again proving the superiority of this design strategy as an artificial protective layer.

**Figure 6:**
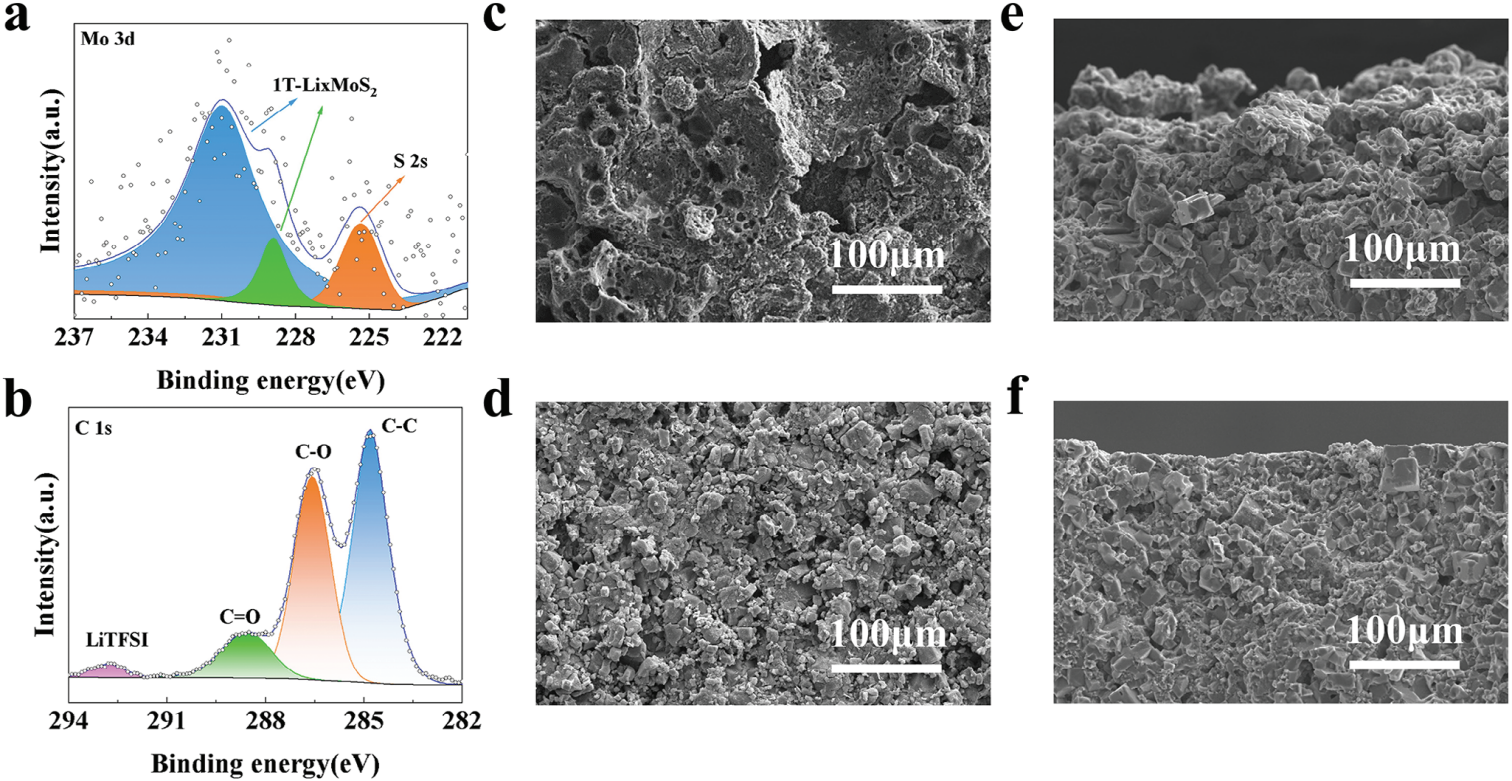
a) Mo 3d XPS spectra and b) C 1s XPS spectra of LATP‐MoS_2_@SP after 50 cycles. Top‐view SEM images of LATP pellets disassembled from c) LFP/LATP/Li and d) LFP/LATP‐MoS_2_@SP/Li cells after 250 cycles. Cross‐sectional SEM image of LATP pellets disassembled from e) LFP/LATP/Li and f) LFP/LATP‐MoS_2_@SP/Li cells after 80 cycles.

We performed XPS analysis on the LATP‐MoS_2_@SP surface after 50 cycles to gain deeper insight into the protection mechanism of the MoS_2_@SP modified layer. In the Mo 3d spectra, the blue and green peaks represent the 1T‐Li*_x_* MoS_2_, and the orange peak represents the peaks of S 2s in the 1T‐Li*_x_* MoS_2_, in addition to no other peaks are detected, indicating that the 2H‐MoS_2_ on the LATP surface is converted to 1T‐Li*_x_* MoS_2_ and gradually tends to be stabilized (**Figure** **6**a). In our previous report, the phase transition of MoS_2_ occurred during cycling.^[^ ^30^ ^]^ Instead, this work produces 1T‐Li*_x_* MoS_2_ before cycling, and the performance of the cells is greatly improved. This is due to the fact that during cycling, the reaction and phase transition of MoS_2_ also destroys the protective layer, leading to changes and cracks in the electrolyte. Instead, the metallic phase 1T‐Li*_x_* MoS_2_ generated before cycling effectively avoids the phase transition reaction that may occur during cycling, allowing the ion transport at the interface to be improved and maintained, which is more conducive to long‐term cycling stability. The C 1s spectra after cycling point C–O peak (286.5 eV) and C═O (288.6 eV) peak, indicating that the PEO is stably present at the Li/LATP interface before and after cycling (Figure 6b), which contributes to the adsorption of more Li‐ion at the interface, and facilitates the interfacial contact. SEM was further employed to verify the modification of the interface by the protective layer, and we tested the morphology of the LATP surface after 250 cycles of the full cells. The disassembled LATP pellet from the LFP/LATP/Li cells exhibits a large number of holes and cracks on the surface after cycling, which impede the ionic transfer and result in the failure of the cells (Figure 6c). However, the LATP‐MoS_2_@SP pellet disassembled from LFP/LATP‐MoS_2_@SP/Li cells exhibits a complete grain shape and no large holes even after 250 cycles (Figure 6d). Meanwhile, Figure 6e,f shows the morphology of the LATP cross‐section after 80 cycles of the full cells, and the surface of the full cells assembled with bare LATP reveals an uneven and jagged shape after cycling, reflecting the poor interfacial contact with Li‐metal. In comparison, the full cells assembled with LATP‐MoS_2_@SP have a flat surface with intact grain structure after cycling, proving intimate and stable interfacial contacts, which is consistent with the results of symmetric cells. From the DFT calculations and the SEM results after cycling, it can be seen that the metallic 1T‐Li*_x_* MoS_2_ can effectively improve the interfacial contact and ionic migration, while the LATP pellets in direct contact with Li‐metal will produce a large number of black reduction products on the surface during cycling due to poor interfacial contact and slow ionic migration, leading to the fracture of the LATP, which further leads to the failure of the cell. Based on the above analysis, the cells assembled with LATP‐MoS_2_@SP have better performance during cycling.

## 3 Conclusion

In summary, we successfully grew large‐area 2H‐MoS_2_ films on the LATP surface by CVD, followed by the incorporation of SP to realize the transition of MoS_2_ from the 2H phase to the 1T phase, avoiding the phase transition reaction during cycling. Li has a much lower migration energy barrier on the 1T‐Li*_x_* MoS_2_ surface, and when LATP‐MoS_2_@SP is used as the interfacial modified layer of Li/LATP, it has higher cycling stability and rate performance compared to the as‐grown 2H‐MoS_2_. This also resulted in the presence of a protective layer at the interface, which allows 1T‐Li*_x_* MoS_2_ to adsorb more Li‐ion, effectively improving the interfacial contact and ion transfer kinetics. The phase transformation method of MoS_2_ used in this work can be extended to general fields such as energy storage, devices, catalysis, etc. More importantly, this study provides universal design ideas for interfacial modification that can be used in SSEs to achieve high‐performance ASSLBs.

## 4 Experimental Section

### Preparation of LATP Solid Electrolyte and SP

Li_1.3_Al_0.3_Ti_1.7_(PO_4_)_3_ (LATP) pellets were prepared by solid‐phase synthesis method, and all the chemicals were of analytical grade, which was purchased from Sinopharm Chemical Reagent Co. and Shanghai Aladdin Biochemical Technology Co. Li_2_CO_3_, Al_2_O_3_, TiO_2_, NH_4_H_2_PO_4_ were first added into corundum ball mill jar according to a certain stoichiometric proportion, in which Li_2_CO_3_ was added at an excess of 10%, and then mixed well and evenly in anhydrous ethanol. The well‐mixed drug was dried in an oven at 180 °C for 10 days, ground, and calcined at 700 °C for 4 h to obtain a uniform precursor powder. An appropriate amount of polyvinyl alcohol with a mass fraction of 5% was added dropwise to the precursor powder and fully ground again. The homogeneously ground powder was weighed 0.2 g at a single time, put into a 12 mm diameter cylindrical tablet press mold, and pressed into round tablets under a uniaxial pressure of 200 MPa. The discs were held at 550 °C for 4 h, and then calcined at 900 °C for 6 h. After the calcination procedure, the LATP tablets were naturally cooled to room temperature. The LATP sheets were sanded and polished using 2000 mesh sandpaper, and then ultrasonically cleaned in anhydrous ethanol and distilled water to obtain a clean, smooth, and flat LATP substrate. 0.25 g of PEO (Mv = 600 000) and 0.0906 g of lithium bis(trifluoromethanesulfonyl)imide (LiTFSI) with a molar ratio of EO/Li of 18:1 were dissolved in anhydrous acetonitrile on a stirring table at 60 °C for 24 h with continuous stirring, and finally SP liquid precursors were formed.

### Preparation of LATP‐MoS_2_ and LATP‐MoS_2_@SP

MoS_2_ was grown on the surface of LATP sheets by chemical vapor deposition (CVD), and the growth experiments were carried out in an ALD‐PE‐CVD dual‐temperature zone tube furnace. The drug was placed in an alumina porcelain boat, sublimation sulfur powder was placed in the upstream temperature zone of the tube furnace, and a homogeneous mixture of MoO_3_ and NaCl powder was placed in the downstream temperature zone, in which NaCl was used as an auxiliary agent to assist the evaporation of the MoO_3_ powder, and the LATP sheet was covered horizontally on the downstream porcelain boat at a horizontal distance of 18–20 mm from the drug. The quartz tubes were filled and deflated three times using pure Ar as the carrier gas to clean the air and maintain the tube. The air was cleaned, and the pressure inside the tube was maintained at normal ambient atmospheric pressure with a carrier gas flux of 400 sccm. The heating program was initiated, and the growth temperature in the upstream sulfur source temperature zone was 200 °C and the growth temperature in the downstream molybdenum source temperature zone was 700 °C and the growth time was 2 h. Finally, natural cooling to room temperature was performed to obtain the LATP‐MoS_2_. For Li/Li symmetric cells, growing twice to get the effect of two‐sided modification was needed. Then the SP liquid precursor solution was quantitatively drop‐coated onto the LATP‐MoS_2_ surface using a pipette gun and subsequently placed in a vacuum drying oven at 60 °C to evaporate the acetonitrile solvent to obtain LATP‐MoS_2_@SP. For Li/Li symmetric cells, drop‐coating twice to get the effect of two‐sided modification was also needed.

### CR2032 Button Cell Assembly

The CR2032 button cells were assembled in a glove box filled with high‐purity argon gas (H_2_O, O_2_ <0.01 ppm). Li/Li symmetric cells were assembled in the order of negative shell, nickel foam, Li‐metal flake, electrolyte, Li‐metal flake, nickel foam, and positive shell. The LFP/Li full cells were assembled in the order of negative shell, nickel foam, Li‐metal flake, electrolyte, LFP, stainless steel sheet, and positive shell. Among them, the MoS_2_@SP protective layer was designed only at the interface between the electrolyte and Li‐metal. Between the electrolyte and the LFP, the 5 µL of liquid electrolyte (1 m LiPF_6_ in EC:DMC = 1:1) to obtain good interfacial contact was added, and then after that, the cell was pressed in the hydraulic press using the appropriate pressure to make the cell structure stable. The cathode active material (LiFePO_4_), conductive carbon black, and binder PVDF were mixed in *N*‐methylpyridinium alkanone (NMP) in a ratio of 6:2:2 and stirred for 10 h, and then coated on the collector aluminum foil. It was dried at 80 °C for 8 h in the blast air and then transferred to a vacuum drying oven at 100 °C for 10 h in a vacuum drying oven until the solvent was completely evaporated. Subsequently, a slicer was used to cut the slices into small discs with a diameter of 10 mm, which were placed in a glove box after drying. The amount of active substance loaded on each slice ranged from 0.67–0.97 mg.

### Characterization and Electrochemical Measurement

The structure of the samples was characterized by a powder X‐ray diffraction (XRD) pattern using a D8‐FOCUS X‐ray diffractometer with CuK_α1_ radiation. The morphology and element analysis were acquired by a SU80101 field scanning electron microscope (SEM) and energy dispersive spectroscopy (EDS). To get the information about the element on the surface of samples, X‐ray photoelectron spectroscopy (XPS) was carried out by Thermo Scientific K‐Alpha+ using Al Kα X‐rays source (1486.6 eV). Raman spectra were measured by Raman spectrometer (Alpha 300‐R) with laser excitation at 532 nm. The electrochemical impendence spectrum (EIS) was carried by a Zennium X electrochemical workstation in the frequency range from 10 MHz to 1 Hz with a 40 mV amplitude. CHI760E was carried out to measure cyclic voltammetry (CV) from 2.7 to 4.2 V with a scan rate of 0.5 mV s^−1^. All cells were conducted on a Wuhan Land battery tester. All of these electrochemical measurements were carried at the temperature of 60 °C.

### Computational Methods

The Vienna Ab Initio Simulation Package (VASP)^[^ ^31^ ^]^ was employed to perform all the spin‐polarized density functional theory (DFT) calculations within the generalized gradient approximation (GGA) in the PBE formulation.^[^ ^32^ ^]^ The projected augmented wave (PAW) potentials were chosen to describe the ionic cores and take valence electrons into account using a plane wave basis set with a kinetic energy cutoff of 450 eV.^[^ ^33^ ^]^ Partial occupancies of the Kohn−Sham orbitals were allowed using the Methfessel–Paxton smearing method and a width of 0.10 eV. The electronic energy was considered self‐consistent when the energy change was smaller than 10^−5^ eV. A geometry optimization was considered convergent when the residual forces were smaller than 0.05 eV Å^−1^. The transition state of an elementary reaction step was located by the nudged elastic band (NEB) method.^[^ ^34^ ^]^ In the NEB method, the path between the reactant(s) and product(s) was discretized into a series of five structural images. The intermediate images were relaxed until the perpendicular forces were smaller than 0.05 eV Å^−1^. Finally, the adsorption energies (*E*_ads_) were calculated as *E*_ads_ = *E*_total_–*E*_ad_–*E*_sub_, where *E*_total_, *E*_ad_, and *E*_sub_ were the total energies of the optimized adsorbate/substrate system, the adsorbate in the structure, and the clean substrate, respectively.

## Conflict of Interest

The authors declare no conflict of interest.

## Supporting information

Supporting Information

## Data Availability

The data that support the findings of this study are available from the corresponding author upon reasonable request.

## References

[1] J. Xiao , F. Shi , T. Glossmann , C. Burnett , Z. Liu , Nat. Energy 2023, 8, 329.

[2] a) S. Han , P. Wen , H. Wang , Y. Zhou , Y. Gu , L. Zhang , Y. Shao‐Horn , X. Lin , M. Chen , Nat. Mater. 2023, 22, 1515; 37845320 10.1038/s41563-023-01693-z

[3] a) P. Wu , W. Zhou , X. Su , J. Li , M. Su , X. Zhou , B. W. Sheldon , W. Lu , Adv. Energy Mater. 2023, 13, 2203440;

[4] a) J. Liu , H. Yuan , H. Liu , C. Z. Zhao , Y. Lu , X. B. Cheng , J. Q. Huang , Q. Zhang , Adv. Energy Mater. 2021, 12, 2100748;

[5] Y. Xiao , Y. Wang , S. H. Bo , J. C. Kim , L. J. Miara , G. Ceder , Nat. Rev. Mater. 2019, 5, 105.

[6] a) J. Zhu , J. Zhao , Y. Xiang , M. Lin , H. Wang , B. Zheng , H. He , Q. Wu , J. Y. Huang , Y. Yang , Chem. Mater. 2020, 32, 4998;

[7] H. Wan , Z. Wang , W. Zhang , X. He , C. Wang , Nature 2023, 623, 739. 37880366 10.1038/s41586-023-06653-w

[8] L. Hu , J. Wang , K. Wang , Z. Gu , Z. Xi , H. Li , F. Chen , Y. Wang , Z. Li , C. Ma , Nat. Commun. 2023, 14, 3807. 37369677 10.1038/s41467-023-39522-1 PMC10300059

[9] Y. K. Liu , C. Z. Zhao , J. Du , X. Q. Zhang , A. B. Chen , Q. Zhang , Small 2023, 19, 2205315.

[10] Q. Zhao , S. Stalin , C. Z. Zhao , L. A. Archer , Nat. Rev. Mater. 2020, 5, 229.

[11] Y. Liu , Q. Sun , Y. Zhao , B. Wang , P. Kaghazchi , K. R. Adair , R. Li , C. Zhang , J. Liu , L. Y. Kuo , Y. Hu , T. K. Sham , L. Zhang , R. Yang , S. Lu , X. Song , X. Sun , ACS Appl. Mater. Interfaces 2018, 10, 31240. 30141900 10.1021/acsami.8b06366

[12] Q. Cheng , A. Li , N. Li , S. Li , A. Zangiabadi , T. D. Li , W. Huang , A. C. Li , T. Jin , Q. Song , W. Xu , N. Ni , H. Zhai , M. Dontigny , K. Zaghib , X. Chuan , D. Su , K. Yan , Y. Yang , Joule 2019, 3, 1510.

[13] X. Hao , Q. Zhao , S. Su , S. Zhang , J. Ma , L. Shen , Q. Yu , L. Zhao , Y. Liu , F. Kang , Y. B. He , Adv. Energy Mater. 2019, 9, 1901604.

[14] L. Luo , F. Zheng , H. Gao , C. Lan , Z. Sun , W. Huang , X. Han , Z. Zhang , P. Su , P. Wang , S. Guo , G. Lin , J. Xu , J. Wang , J. Li , C. Li , Q. Zhang , S. Wu , M. S. Wang , S. Chen , Nano Res. 2023, 16, 1634.

[15] J. Y. Liang , X. X. Zeng , X. D. Zhang , T. T. Zuo , M. Yan , Y. X. Yin , J. L. Shi , X. W. Wu , Y. G. Guo , L. J. Wan , J. Am. Chem. Soc. 2019, 141, 9165. 31141357 10.1021/jacs.9b03517

[16] L. Zhu , Y. Wang , Y. Wu , W. Feng , Z. Liu , W. Tang , X. Wang , Y. Xia , Adv. Funct. Mater. 2022, 32, 2201136.

[17] L. Luo , Z. Sun , H. Gao , C. Lan , X. Huang , X. Han , P. Su , Z. Zhang , C. Li , W. Huang , Q. Wei , Q. Zhang , M. S. Wang , S. Chen , Adv. Energy Mater. 2023, 13, 2203517.

[18] Z. Li , W. Zheng , G. Lu , M. Li , D. Tang , Q. Zhao , Y. Wang , C. Xu , R. Wang , Adv. Funct. Mater. 2024, 34, 2309751.

[19] S. Wang , D. Zhang , B. Li , C. Zhang , Z. Du , H. Yin , X. Bi , S. Yang , Adv. Energy Mater. 2018, 8, 1801345.

[20] M. Acerce , D. Voiry , M. Chhowalla , Nat. Nanotechnol. 2015, 10, 313. 25799518 10.1038/nnano.2015.40

[21] J. Fu , P. Yu , N. Zhang , G. Ren , S. Zheng , W. Huang , X. Long , H. Li , X. Liu , Energy Environ. Sci. 2019, 12, 1404.

[22] C. Xu , B. Sun , T. Gustafsson , K. Edström , D. Brandell , M. Hahlin , J. Mater. Chem. A 2014, 2, 7256.

[23] H. Tang , J. Wang , H. Yin , H. Zhao , D. Wang , Z. Tang , Adv. Mater. 2015, 27, 1117. 25529000 10.1002/adma.201404622

[24] K. Leng , Z. Chen , X. Zhao , W. Tang , B. Tian , C. T. Nai , W. Zhou , K. P. Loh , ACS Nano 2016, 10, 9208. 27636565 10.1021/acsnano.6b05746

[25] J. Xiao , X. Wang , X. Q. Yang , S. Xun , G. Liu , P. K. Koech , J. Liu , J. P. Lemmon , Adv. Funct. Mater. 2011, 21, 2840.

[26] D. Y. W. Yu , C. Fietzek , W. Weydanz , K. Donoue , T. Inoue , H. Kurokawa , S. Fujitani , J. Electrochem. Soc. 2007, 154, A253.

[27] J. Xiao , Q. Li , Y. Bi , M. Cai , B. Dunn , T. Glossmann , J. Liu , T. Osaka , R. Sugiura , B. Wu , J. Yang , J. G. Zhang , M. S. Whittingham , Nat. Energy 2020, 5, 561.

[28] Z. Chen , G. T. Kim , J. K. Kim , M. Zarrabeitia , M. Kuenzel , H. P. Liang , D. Geiger , U. Kaiser , S. Passerini , Adv. Energy Mater. 2021, 11, 2101339.

[29] a) X. Hao , K. Chen , Y. Tang , X. Zhong , K. Cai , J. Alloys Compd. 2023, 942, 169064;

[30] C. Huang , Z. Li , S. Duan , S. Xie , S. Yuan , S. Hou , G. Cao , H. Jin , J. Power Sources 2022, 536, 231491.

[31] a) G. Kresse , J. Furthmüller , Comput. Mater. Sci. 1996, 6, 15;

[32] J. P. Perdew , K. Burke , M. Ernzerhof , Phys. Rev. Lett. 1996, 77, 3865. 10062328 10.1103/PhysRevLett.77.3865

[33] a) G. Kresse , D. Joubert , Phys. Rev. B 1999, 59, 1758;

[34] G. Henkelman , B. P. Uberuaga , H. Jónsson , J. Chem. Phys. 2000, 113, 9901.

