# Supplementary material for: Designing the Interface Layer of Solid Electrolytes for All‐Solid‐State Lithium Batteries
Source: Adv Sci (Weinh). 2024 Jun 3;11(29):2401453. doi: 10.1002/advs.202401453 (PMC11304316; doi:10.1002/advs.202401453)
Supplement: Supplementary file 1 — Supporting Information [file ADVS-11-2401453-s001.docx]

Supporting Information

Designing the Interface Layer of Solid Electrolytes for All-solid-state Lithium Batteries

*Qian Xia^a^, Shuoguo Yuan^a^*, Qiang Zhang^a^, Can Huang^a^, Jun Liu^b^, Hongyun Jin^a^**

^a^Faculty of Materials Science and Chemistry, China University of Geosciences, Wuhan 430074, China

^b^Guangdong Provincial Key Laboratory of Advanced Energy Storage Materials, School of Materials Science and Engineering, South China University of Technology Guangzhou 510641, China


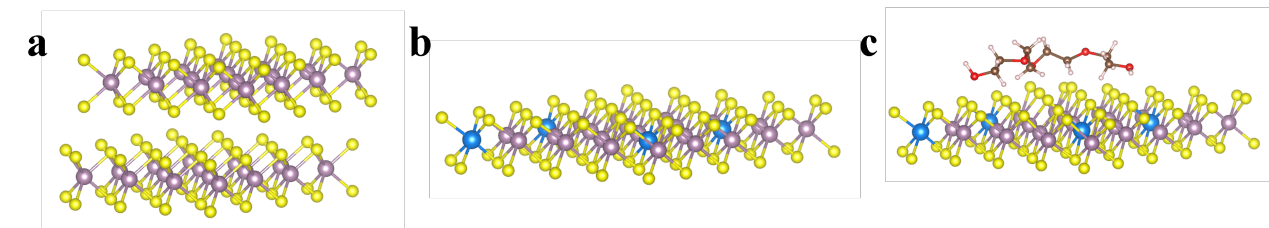


Figure S1. Structure of (a) 2H-MoS_2_ and (b) 1T-Li*_x_*MoS_2_. (c) 1T-Li*_x_*MoS_2_/PEO.


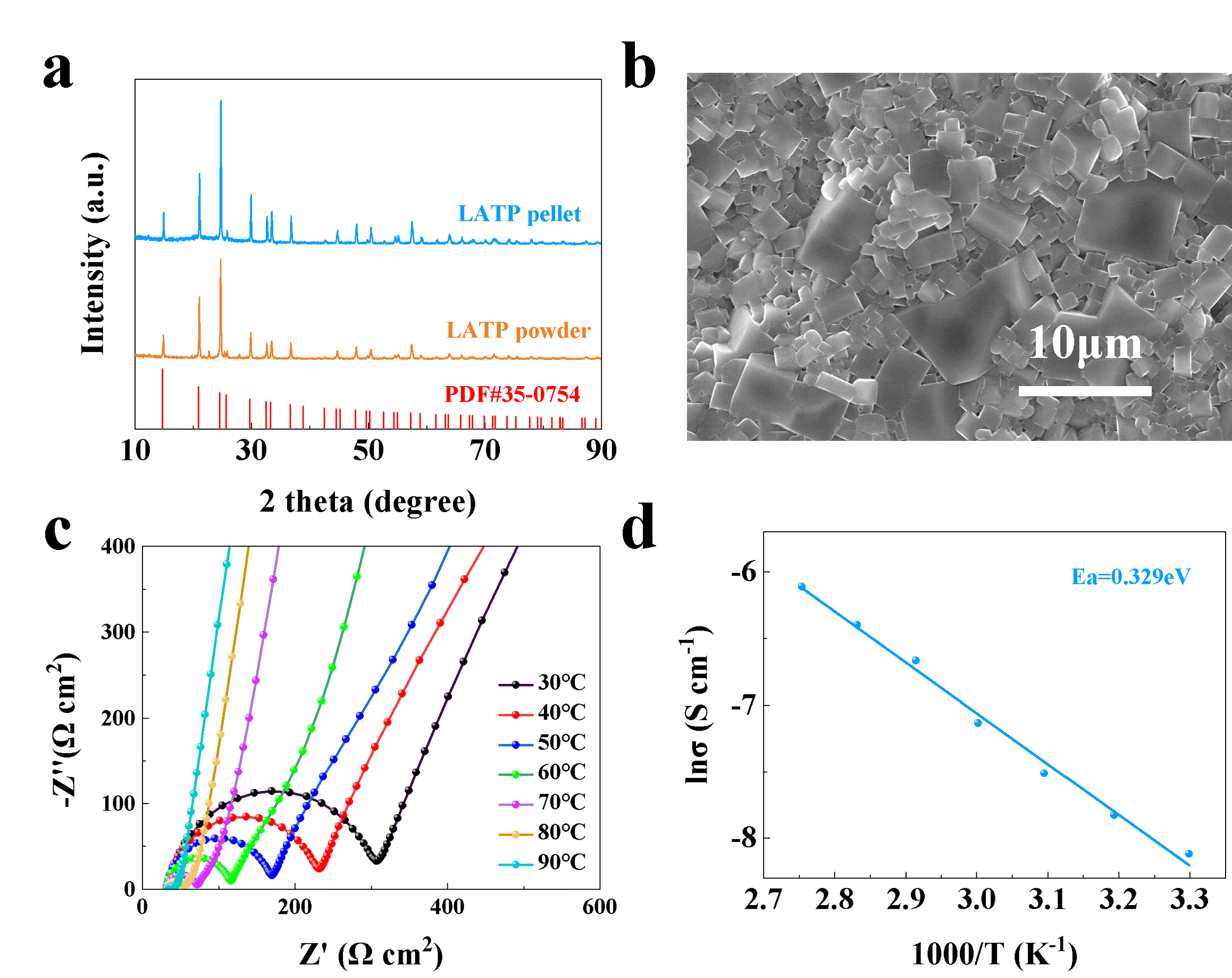


Figure S2. (a) XRD patterns of LATP powder and LATP pellet, which match well with the LiTi_2_(PO_4_)_3_ (PDF# 35-0754). (b) Top-view SEM image of LATP pellet. (c) Electrochemical impedance spectra of the Au/LATP/Au at various temperature. (d) Arrhenius plot of the LATP pellets at various temperatures, where the activation energy is 0.329 eV.


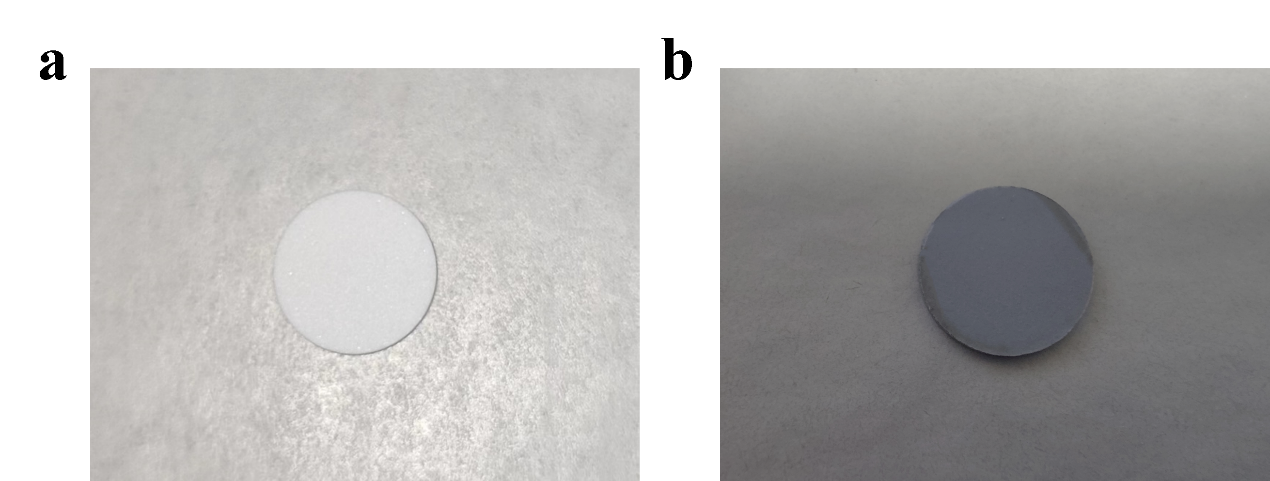


Figure S3. LATP pellet (a) before and (b) after growth.


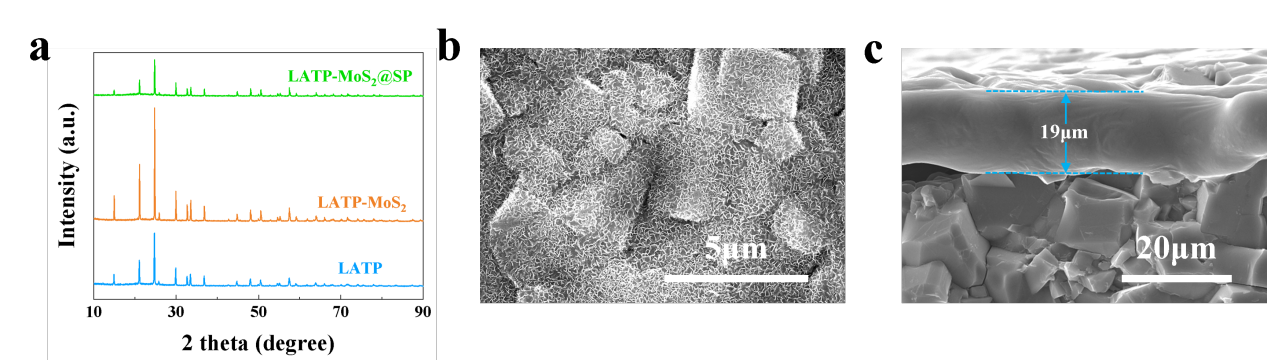


Figure S4. (a) XRD patterns of LATP, LATP-MoS_2_, LATP-MoS_2_@SP, no significant change in comparison. (b)Top-view SEM image of LATP-MoS_2_@SP, reduced view corresponding to Figure 3a. (c) Cross-sectional SEM image of LATP-MoS_2_@SP, where MoS_2_@SP thickness is 19 μm.


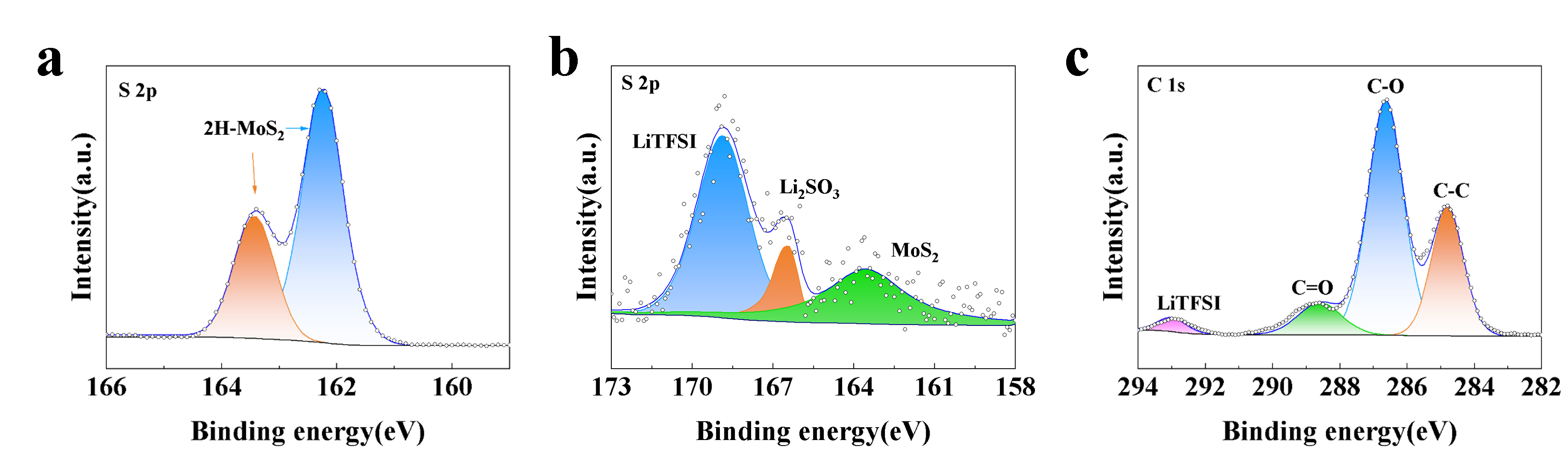


Figure S5. S 2p XPS spectra of (a) LATP-MoS_2_ and (b) LATP-MoS_2_@SP. (c) C 1s spectra of LATP-MoS_2_@SP.


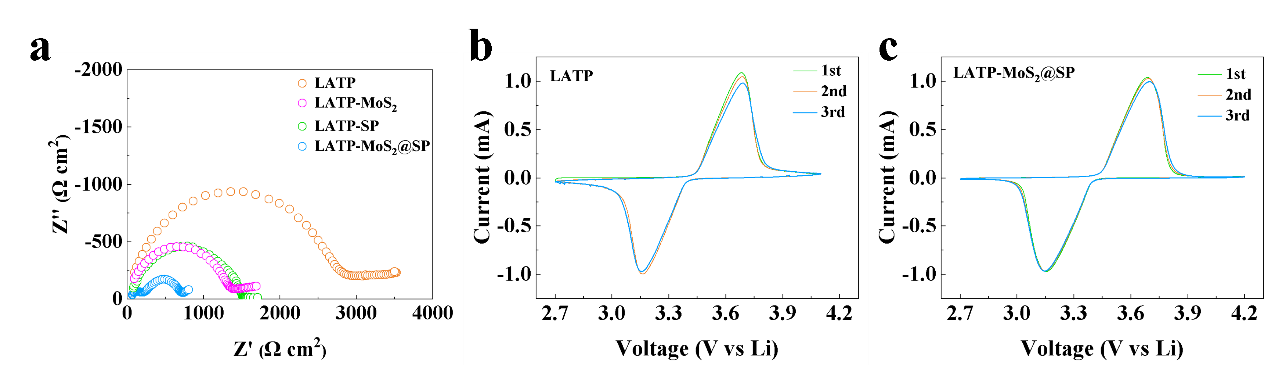


Figure S6. (a) EIS of Li/LATP/Li, Li/LATP-MoS_2_/Li, Li/LATP-SP/Li, Li/LATP-MoS_2_@SP/Li cells at 60 ℃. The CV curves of (b) LFP/LATP/Li and (c) LFP/LATP-MoS_2_@SP/Li cells.


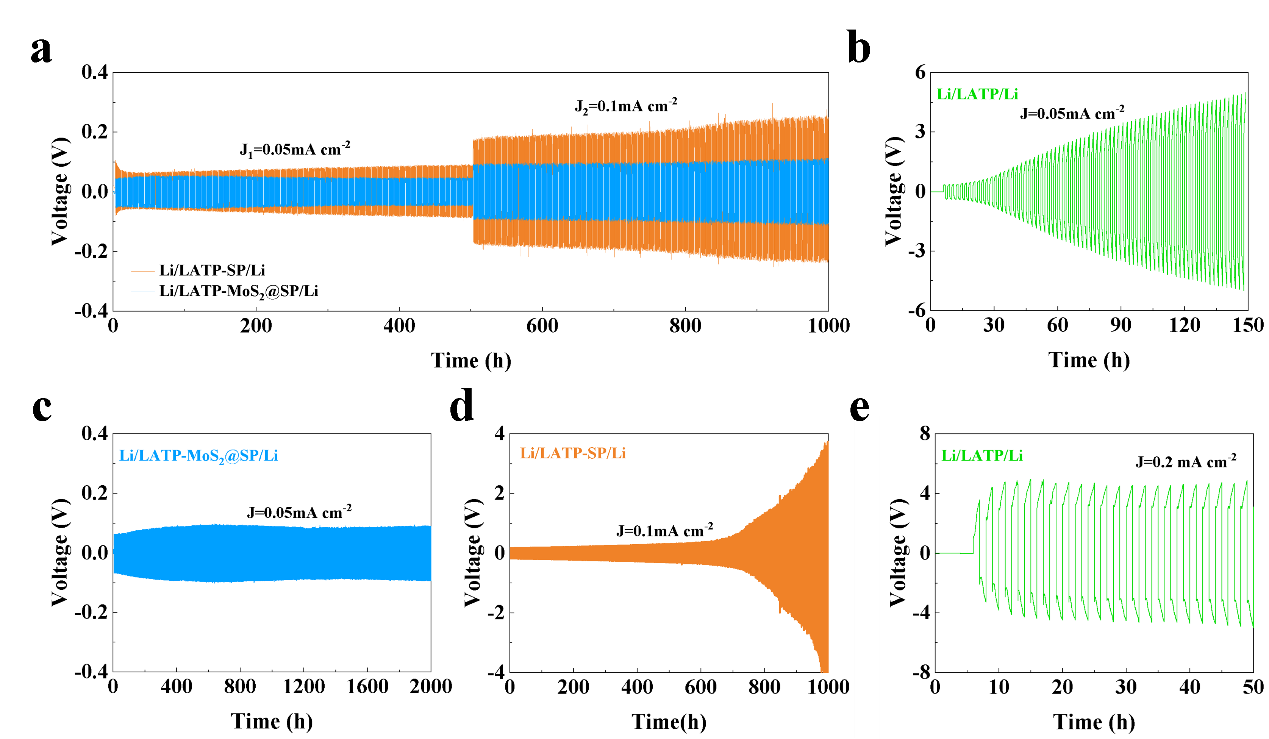


Figure S7. (a) Constant current stripping and plating of Li/LATP-SP/Li and Li/LATP-MoS_2_@SP/Li cells, current density of 0.05 mA cm^-2^ for 0 to 500 h and 0.1 mA cm^-2^ for 500 h to 1000 h. Constant current stripping and plating of (b) Li/LATP/Li cells (c) Li/LATP-MoS_2_@SP/Li cells at 0.05 mA cm^-2^. (d) Constant current stripping and plating of Li/LATP- SP/Li cells at 0.1 mA cm^-2^. (e) Constant current stripping and plating of Li/LATP /Li cells at 0.2 mA cm^-2^.


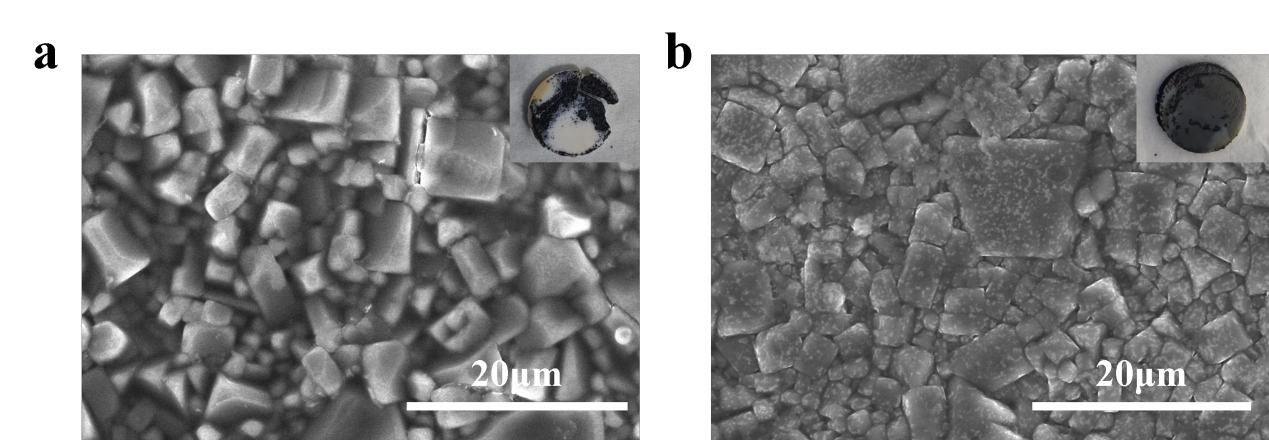


Figure S8. Top-view SEM images of LATP after 50 h of cycling of (a) Li/LATP/Li and (b) Li/LATP-MoS_2_@SP/Li cells at 0.05 mA cm^-2^.


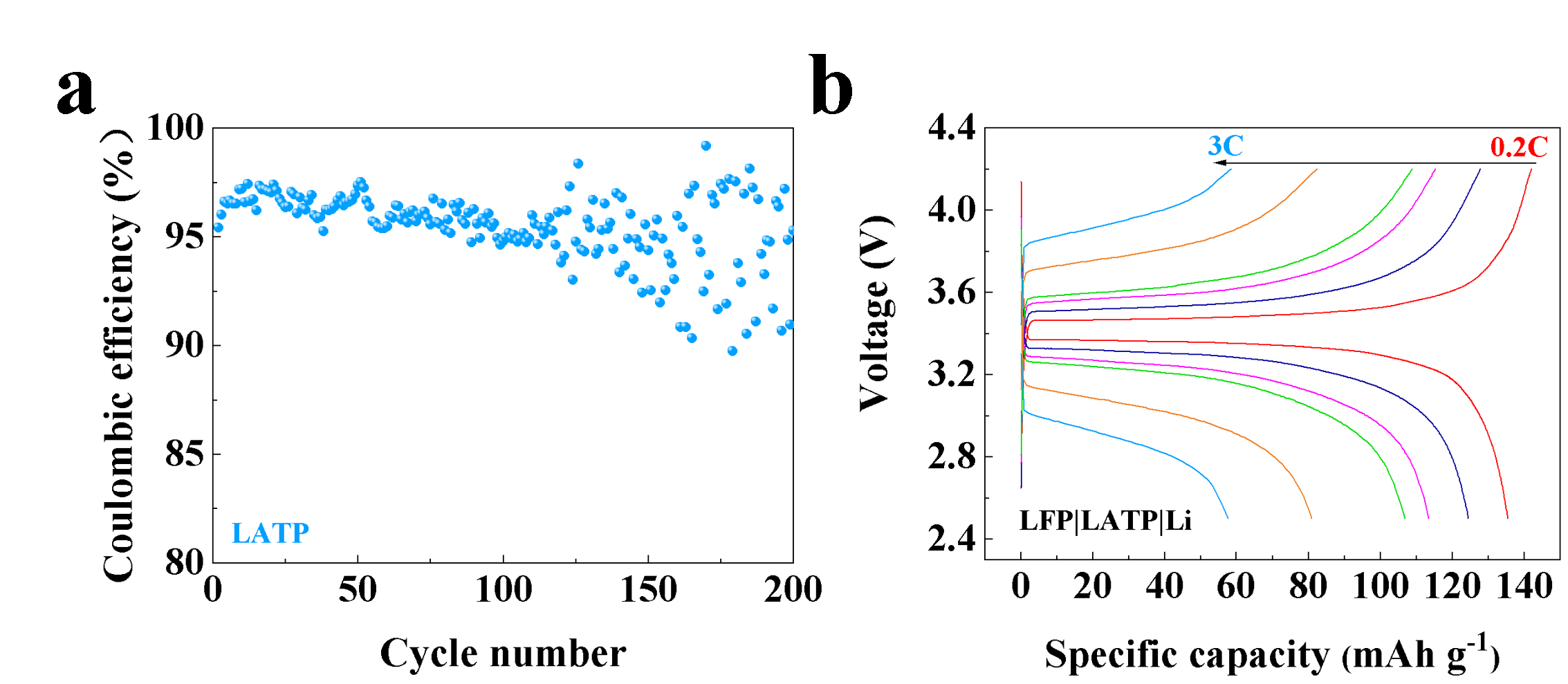


Figure S9. (a) Coulombic efficiency of LFP/LATP/Li cells within 200 cycles. (b) The charging/discharging profiles with different rates in LFP/LATP/Li cells.


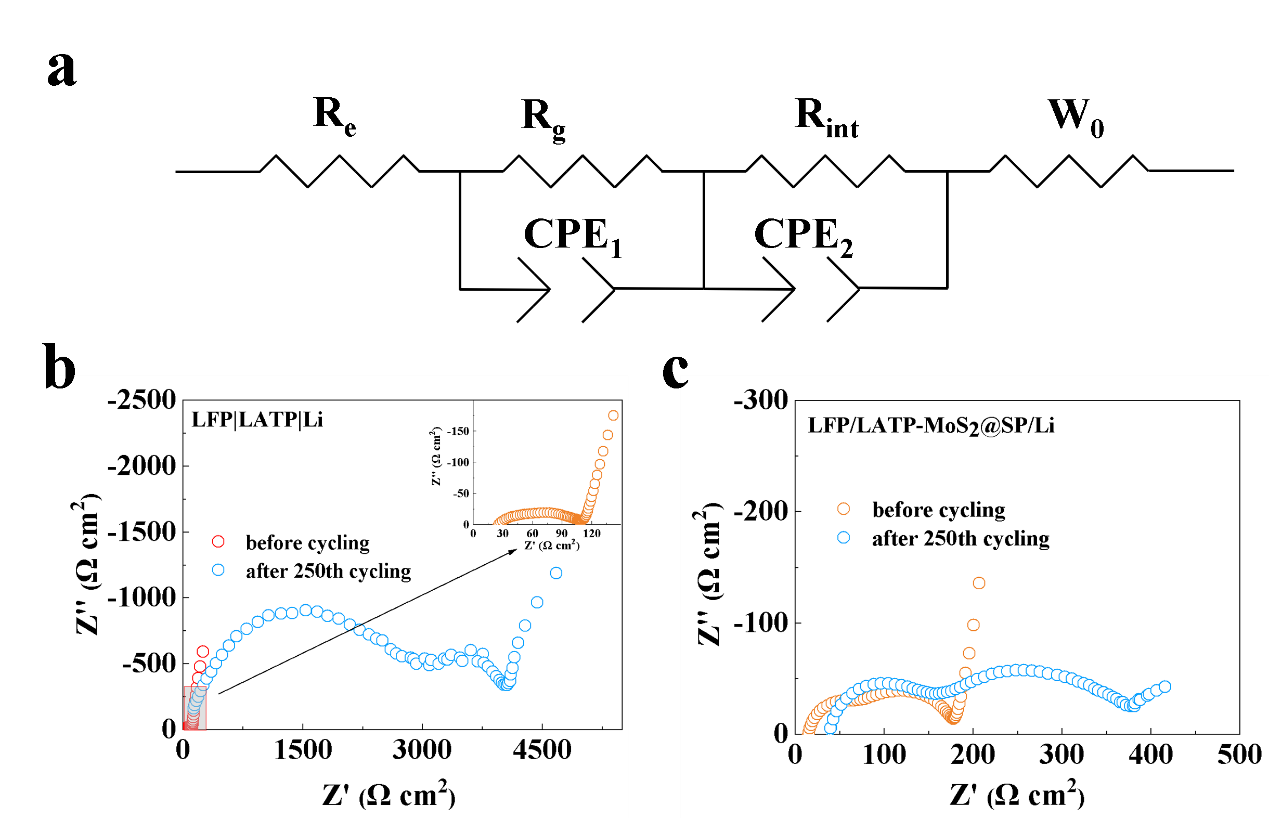


Figure S10. (a) The fitting equivalent circuit model. R_e_ is the bulk resistance of LATP. R_g_ and CPE_1_ are the grain boundary resistance in LATP and constant phase element (CPE) for the inter-grain capacitance, respectively. R_int_ and CPE_2_ are interfacial resistance and the CPE which represents interfacial capacitance at the electrolyte/lithium interface, respectively.^[1]^ (b) LFP/LATP/Li and (c) LFP/LATP-MoS_2_@SP/Li cells before cycling and after 250 cycles.


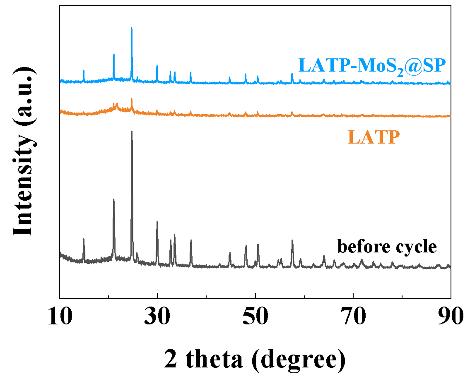


Figure S11. XRD patterns of pre-cycling as well as bare LATP and LATP-MoS_2_@SP after 250 cycles.

Table S1. Comparison of long-term cycle performance with previously reported results of LATP solid electrolyte.

| Interfacial modification | Rate | Temperature | Cycle number | Capacity retention | Ref. |
| --- | --- | --- | --- | --- | --- |
| BaTiO_3_ /(P[VDF-TrFE-CTFE] | 0.5 | RT | 250 | 78.6 | ^[2]^ |
| ZnO | 0.1 | RT | 200 | 88 | ^[3]^ |
| 2-(TMS)PTM | 0.2 | 30 | 300 | 86.2 | ^[4]^ |
| PVDF-TrFE | 0.5 | 20 | 112 | 80 | ^[5]^ |
| PEO-PAN | 0.5 | 60 | 120 | 89 | ^[6]^ |
| KANF@SE | 0.1 | 30 | 180 | 95 | ^[1]^ |
| Al-LiF | 0.5 | 30 | 350 | 83.2 | ^[7]^ |
| PVDF-HFP-LiTFSI | 1 | 60 | 300 | 97.2 | ^[8]^ |
| IL@SPF | 0.4 | 60 | 215 | 77.8 | ^[9]^ |
| PEO | 0.3 | 60 | 100 | 97.3 | ^[10]^ |
| IL@SPF | 0.1 | 60 | 100 | 66.1 | ^[11]^ |
| I-SN | 0.5 | RT | 100 | 93.17 | ^[12]^ |
| MoS_2_@SP | 1 | 60 | 400 | 86 | This work |

References

[1] W. Kong, Z. Jiang, Y. Liu, Q. Han, L. X. Ding, S. Wang, H. Wang, *Adv. Funct. Mater.* 2023, 33, 2306748.

[2] T. Gu, L. Chen, Y. Huang, J. Ma, P. Shi, J. Biao, M. Liu, W. Lv, Y. He, *Energy Environ. Mater.* 2023, 6, e12531.

[3] X. Hao, Q. Zhao, S. Su, S. Zhang, J. Ma, L. Shen, Q. Yu, L. Zhao, Y. Liu, F. Kang, Y. B. He, *Adv. Energy Mater.* 2019, 9, 1901604.

[4] Y. Xu, M. Tian, Y. Rong, C. Lu, Z. Lu, R. Shi, T. Gu, Q. Zhang, C. Jin, R. Yang, *J. Colloid Interface Sci.* 2023, 641, 396.

[5] Z. Chen, G. T. Kim, J. K. Kim, M. Zarrabeitia, M. Kuenzel, H. P. Liang, D. Geiger, U. Kaiser, S. Passerini, *Adv. Energy Mater.* 2021, 11, 2101339.

[6] J. Y. Liang, X. X. Zeng, X. D. Zhang, T. T. Zuo, M. Yan, Y. X. Yin, J. L. Shi, X. W. Wu, Y. G. Guo, L. J. Wan, *J. Am. Chem. Soc.* 2019, 141, 9165.

[7] L. Luo, F. Zheng, H. Gao, C. Lan, Z. Sun, W. Huang, X. Han, Z. Zhang, P. Su, P. Wang, S. Guo, G. Lin, J. Xu, J. Wang, J. Li, C. Li, Q. Zhang, S. Wu, M.-S. Wang, S. Chen, *Nano Res.* 2023, 16, 1634.

[8] L. Pan, S. Sun, G. Yu, X. X. Liu, S. Feng, W. Zhang, M. Turgunov, Y. Wang, Z. Sun, *Chem. Eng. J.* 2022, 449, 137682.

[9] M. Lei, S. Fan, Y. Yu, J. Hu, K. Chen, Y. Gu, C. Wu, Y. Zhang, C. Li, *Energy Storage Mater.* 2022, 47, 551.

[10] Z. Yang, H. Yuan, C. Zhou, Y. Wu, W. Tang, S. Sang, H. Liu, *Chem. Eng. J.* 2020, 392, 123650.

[11] M. Lei, S. Fan, Y. Yu, J. Hu, K. Chen, Y. Gu, C. Wu, Y. Zhang, C. Li, *Energy Storage Mater.* 2022, 47, 551.

[12] Q. Liu, Q. Yu, S. Li, S. Wang, L. Zhang, B. Cai, D. Zhou, B. Li, *Energy Storage Mater.* 2020, 25, 613.
